# Supplementary material for: Lexical simplification benchmarks for English, Portuguese, and Spanish
Source: Front Artif Intell. 2022 Sep 22;5:991242. doi: 10.3389/frai.2022.991242 (PMC9536312; doi:10.3389/frai.2022.991242)
Supplement: Supplementary file 1 [file Data_Sheet_1.zip › appendix.pdf]

1 APPENDIX I: INSTRUCTIONS FOR ANNOTATORS

Below are **N** sentences in English/Spanish/Portuguese, in each sentence there is a word marked in bold. Your task is to write, in the space below each sentence, single word that has the same meaning as the one marked, but is easier to understand. For example, in the sentence ”At the same time, the rate of decline against the dollar was **attenuated**” the word **attenuated** could be replaced by the easier-to-understand word *decreased*. Write the replacement so that the replacement is valid in the given context. In our example, *decreased* is correct while decrease would not be correct. In that case that it is not possible to replace with a single word, then you can use a more complex substitution. For example in the sentence ”The dresses were **Iranian**”, the word **Iranian** could be replaced by “**from Iran**”. Replacements that involve a gender change with respect to the marked word are also allowed in Spanish and Portuguese (Note that this is not applicable in English).

Note 1: If you cannot find a simpler word then you must write the same complex word in the answer area.

Note 2: You are allowed to use all kinds of lexical reference resources such as dictionaries, thesaurus, etc., whether books or online, to do the task.

WARNING: In this task it is important that you follow the instructions to receive your payment. By completing the task and clicking the purple button ”Send” you affirm that you have read and agree to the conditions of the information and consent form.

Information and Consent Form

The study aims to collect examples of lexical simplification for English/Spanish/Portuguese. The data collected will be used for research purposes only. You will read sentences in which a word considered complex will appear that you should simplify by proposing another word that has the same meaning but is easier to understand. The data collected will be used in a research project and will be provided to researchers who need it. The results of this research may be published in scientific journals or conferences and may be used in subsequent studies. To participate in this experiment you should:

- a) Be a native English/Spanish/Portuguese speaker,
- b) Be at least 18 years old and competent to give consent.
- c) Have read and understood this Information Form that explains the research project,
- d) You agree that the data collected will be used anonymously in the future,
- e) Agree to participate in the research described above.

Thanks for participating!

2 APPENDIX II: EXAMPLE OUTPUTS IN EACH LANGUAGE

Example 1

Context and target: *A local witness said a separate group of attackers* **disguised** *in burgas – the head-to-toe robes worn by conservative Afghan women – then tried to storm the compound.*

Gold: concealed, dressed, hidden, camouflaged, changed, covered, masked, unrecognizable, converted, impersonated

LSBert: **dressed**, **hidden**, hiding, **covered**, buried

TUNER: disguised, **masked**

Example 2

Context and target: *War* **maniacs** *of the South Korean puppet military made another grave provocation to the DPRK in the central western sector of the front on Thursday afternoon.*

Gold: fanatics, crazies, freaks, addicts, fans, lunatics, enthusiasts, fiends, fools, hawks, mongers, nuts, psychos

LSBert: criminals, victims, machines, **freaks**, people

TUNER: **lunatics**, madmans, maniacs

Example 3

Context and target: *That prompted the military to* **deploy** *its largest warship, the BRP Gregorio del Pilar, which was recently acquired from the United States.*

Gold: send, post, use, position, employ, extend, launch, let loose, organize, redistribute, release, send out, set up, situate, station

LSBert: **launch**, dispatch, **use**, develop, **employ**

TUNER: deploy

Table 1. Instances from the English part of the dataset together with the output of TUNER and LSBert systems for English. The suggested replacements in the gold data are presented in descending order with respect to the number of people who suggested them. The most frequently suggested ones are underlined (ties are possible). Suggestions by the systems that are present in the gold data are shown in bold.

Example 1

Context and target: *Las bancas (exedras) y balaustradas fueron hechas de mármol y el piso de granito, también habían cuatro fuentes de agua, faroles de bronce y jardines de flores que encantaban mucho a los* **transeúntes**

Gold: peatones, caminantes, paseantes, pasantes, personas que caminaban por ahí, viandantes, pasajeros, pedestres, personas con barandal, viajantes, las personas que pasaban

LSBert: turistas, vecinos, **pasajeros**, ciudadanos, visitantes

TUNER: transeúntes

Example 2

Context and target: *Conforme avanzaba el debate en el Congreso de Filadelfia, Lee iba asumiento una posición más favorable a la independencia total y no sólo a la autonomía del Imperio Británico, su* **convicción** *de la necesidad de la independencia logró convencer a delegados de otras colonias e incluso persuadió a sus propios electores de Virginia, temerosos que Lee pudiera llegar demasiado lejos.*

Gold: creencia, seguridad, certeza, convencimiento, ideal, fé, persuación, fuerte creencia, idea

LSBert: **idea**, **creencia**, sentido, sentimiento, experiencia

TUNER: **certeza**, convicción

Example 3

Context and target: *La Abadía de Fontenay es una abadía francesa de Marmagne (departamento de Côte-d'Or), uno de los* **monasterios** *más emblemáticos de toda la arquitectura cisterciense.*

Gold: conventos, templos, las iglesias, ecierro religioso, claustros, lugares

LSBert: **lugares**, edificios, sitios, puntos, elementos

TUNER: monasterios, **conventos**

Table 2. Instances from the Spanish part of the dataset together with the output of TUNER and LSBert systems for Spanish. The suggested replacements in the gold data are presented in descending order with respect to the number of people who suggested them. The most frequently suggested ones are underlined (ties are possible). Suggestions by the systems that are present in the gold data are shown in bold.

Example 1
Context and target: *Quem não conseguir* **esgotar** *o armazenamento de diesel puro não pode misturar com o b2 porque o produto ficaria fora de especificação.*
Gold: acabar, esvaziar, acabar com, gastar, consumir, diminuir, zerar
LSBert: recuperar, terminar, **acabar**, completar, fazer
TUNER: **consumir**, esgotar
Example 2
Context and target: *Naquele país a ave é considerada uma* **praga**.
Gold: peste, epidemia, maldição, doença, desgraça, tragédia, infestação
LSBert: ameaça, **doença**, droga, delas, espécie
TUNER: praga, **peste**
Example 3
Context and target: *Autores de furto estariam* **migrando** *para o roubo.*
Gold: mudando, passando, deslocando-se, indo, se direcionando, deslocando, se mudando
LSBert: **indo**, trabalhando, saindo, entrando, voltando
TUNER: migrando, emigrando

Table 3. Instances from the Portuguese part of the dataset together with the output of TUNER and LSBert systems for Portuguese. The suggested replacements in the gold data are presented in descending order with respect to the number of people who suggested them. The most frequently suggested ones are underlined (ties are possible). Suggestions by the systems that are present in the gold data are shown in bold.
